# What is the impact of restricted access policy on workplace violence in general hospital? A before-after study in a CHINESE tertiary hospital

**DOI:** 10.1186/s12913-020-05757-7

**Published:** 2020-10-12

**Authors:** Haonan Jia, Ruohui Chen, Lifeng Wei, Gangyu Zhang, Mingli Jiao, Chao Liu, Zhuowa Sha, Shuang Zhou, Yameng Wang, Jingqun Li, Xiaowen Jia, Omar Yacouba Ismael, Jingfu Mao, Qunhong Wu

**Affiliations:** 1 Department of Medical Affairs, The First Affiliated Hospital, Sun Yat-sen University, Guangzhou, China; 2 Department of Health Policy and Hospital Management, School of Public Health, Harbin Medical University, No.157 Baojian Road, Nangang District, Harbin, 150086 Heilongjiang China; 3 Department of Human Resources, The First Affiliated Hospital, Sun Yat-sen University, Guangzhou, China; 4 Heilongjiang Infectious Disease Prevention and Control Hospital, Harbin, China; 5 Institute of Quantitative& Technical Economics, Chinese Academy of Social Science, Beijing, China; 6 Department of Cardiology, No.1 People’s Hospital of Heihe, Heihe, China; 7 Department of General Surgery, No.1 People’s Hospital of Heihe, Heihe, China; 8 Department of Human Resource Management, School of Public Health, Harbin Medical University, Harbin, China; 9 Department of Social Medicine, School of Public Health, Harbin Medical University, Harbin, China

**Keywords:** Workplace violence, Healthcare professionals, Intervention, Restricted access

## Abstract

**Background:**

To evaluate the impact of a restricted access policy on workplace violence in a healthcare setting.

**Methods:**

We surveyed healthcare workers before and after the implementation of a restricted-access policy at a tertiary hospital in north-eastern China. Data were collected in April 2017 and January 2019. Fisher’s exact test were used to compare the difference in workplace violence prevalence between responses to two surveys. Survey 1 (S1) collected data from 345 healthcare professionals who had worked in the inpatient ward for at least 12 months. Survey 2 (S2) included 338 healthcare workers from the same ward who had been employed for more than two years. The effective response rates for the two studies was 79.31 and 83.25%, respectively. All 18 female security guards were included in the investigation in S2.

**Results:**

The prevalence of psychological violence was 62.03% in S1 and 34.62% in S2, the difference in prevalence showing statistical significance (*P* = 0.000), while the prevalence of physical violence was 3.77 and 4.73% respectively, showing no statistical significance (*P* = 0.573). The change in the rate of injury caused by physical violence was also statistically significant at 76.92 and 31.25% (*P* = 0.025), respectively. Security guards were at high risk of workplace violence under the policy. Most healthcare professionals thought this policy ameliorated treatment order, the sense of security, anxiety about workplace violence, and so forth, but one-third of the respondents thought that it caused patient dissatisfaction.

**Conclusion:**

While the restricted access policy may be effective for healthcare professionals in avoiding or dealing with violence, such policy could contribute to new problems regarding the safety of security guards and the potential dissatisfaction of patients. The policy should be further developed to alleviate this phenomenon.

## Background

Workplace violence (WPV) in healthcare settings is a worldwide serious issue [1]. For healthcare professionals, WPV not only negatively impacts their physical and psychological health, [2] but also work performance, work efficiency, work satisfaction, staff retention, and staff morale [3]. Due to the seriousness of these consequences, it is of high practical value to formulate effective interventions against WPV. In 2004, the US Occupational Safety and Health Administration (OSHA) published Guidelines for Preventing Workplace Violence for Healthcare and Social Service Workers and updated in 2016, which provides an outline for healthcare institutions and researchers for conducting interventions on WPV [4]. In general, the strategies could be categorized into 3 levels: 1) social level (e.g. laws and regulations to prevent healthcare professionals from suffering WPV). 2) organizational level (e.g. policy and the environment of an organization designed to encourage WPV reporting, social support and cooperation from the organization, installation of cameras, improvement of security); 3) personal level (e.g. training and education about communication, identification of the precursors of violence, violence minimization, and de-escalation). Studies have indicated that the reporting WPV system [5], equipped with mobile phone or protective equipment [6] is helpful to reduce WPV. Heckemann et.al suggested that staff training could enhance confidence in dealing with WPV, but there is no significant long-term effects in WPV reduction [7]. Also study shows that implementing restructuring environment, improving work organization and staff education simultaneously could reduce WPV [8].

In Chinese general hospitals, more than 50% WPV cases were perpetrated by patient’s families or other visitors [9–11]. Restricted access is an organizational level intervention using security guards and doors to prevent outsiders who may be potential perpetrators from entering wards, especially visitors or patients’ families. The approach has been widely used in some countries. The 2018 Hospital Security Survey conducted in 315 American hospitals indicated every hospital either already has electronic-access control or plans to implement it in the next 24 months, 42% hospitals reported having a visitor-management system [12]. Also, in some developed countries, such as UK and Canada, restricted access is a common measure to protect healthcare workers [13, 14]. As a developing country, some hospitals in India that have sufficient financial capabilities hired guards to control visitors and to handle disputes [15]. However, hospitals in China have just begun to implement this measure. In China, there is no strict regulation of reservation and referral in healthcare service; consequently, people seek treatment on their own accord. Additionally, owing to the shortage of nurses and the Chinese traditional culture of filial piety and support of relatives, family members undertake most of the basic care of patients in hospitals. These conditions can lead to a chaotic environment as there may be many unidentified people coming and going among the staff and patients, potentially threatening the health and safety of the healthcare professionals. Since wards, offices and corridors in hospital buildings were the high-risk place that WPV happens [16], China has enacted ‘Guidance on strengthening the security and protection system construction in hospitals’ in 2013 and ‘Opinions on strictly punishing medical related crimes and maintaining the medical order’ in 2017 [17, 18]. Although these government guidance provide some instructions for Chinese hospitals to improve safety and order, such as stationing a security guard for every 20 beds, installing protective doors and providing security guards in secondary or tertiary hospitals, it is not a mandatory law that hospitals must enforce. It tends to be a kind of support to comfort and relax healthcare professionals as social level intervention, but the impact of these policy is still unknown. Although Chinese hospitals have introduced measures to restrict unidentified outsiders’ access to wards, this may spark new disputes. At present, there has only been limited research evaluating the impacts of the restricted access policy on WPV occurrence.

According to studies of WPV intervention, anti-violence training is the most common strategy employed. Heckerman suggested that training is not necessarily useful in WPV reduction, but it could strengthen confidence in managing WPV in short term [7]. According to Morphet’s classification of WPV interventions, anti-WPV training is a measure of ‘Staff Education’, while restricted access is classified as ‘Environmental Risk Management’. Restricted access is widely used in psychiatric wards [19]. Consequently, research evaluating the impact of the restricted access policy has been conducted mainly in psychiatric wards. Studies have indicated that locked doors in psychiatric hospitals could create safety and security, [20–23] prevent unwelcome visitors and illegal substances from entering the ward, [21, 22, 24, 25] and allow healthcare workers to spend more time on treatment instead of watching the door [22]. However, the main purpose of a restricted access policy in a psychiatric hospital differs from that of a general hospital in that in addition to reducing WPV for healthcare workers and promoting better medical order in wards, it must focus on negating patients’ aggressive behaviours towards themselves and others. In a general hospital setting, restricted access is to protect healthcare professionals from WPV and to maintain better medical order in wards. Owing to the discrepancy of settings and purpose in implementing restricted access, the procedures that have been in use in psychiatric hospital settings cannot be directly applied to general hospitals.

This study aims to find out the impacts of a restricted access policy in controlling WPV in a general hospital setting, as measured by changes in the prevalence of and feelings about WPV before and after implementation.

## Methods

### Study design

We conducted a before-after study including two surveys in a tertiary hospital in north-eastern China: S1(before restricted access) and S2(after restricted access). The participants are all the healthcare professionals and security guards that meet the standard in this hospital.

### Restricted access policy

In 2017, the restricted access policy was recommended in related documents of Provincial government, which aims to maintain order and to enhance security thus to reduce WPV in healthcare professionals working area. In October 2017, the subject hospital activated a restricted access policy in the inpatient ward building but not in the administration and outpatient buildings. This policy was made to prevent potential perpetrators form entering wards. Unrelated persons, such as expressman, salesman, visitors without permission by hospital, would not be permitted to go inside the wards.

**Fig. 1:**
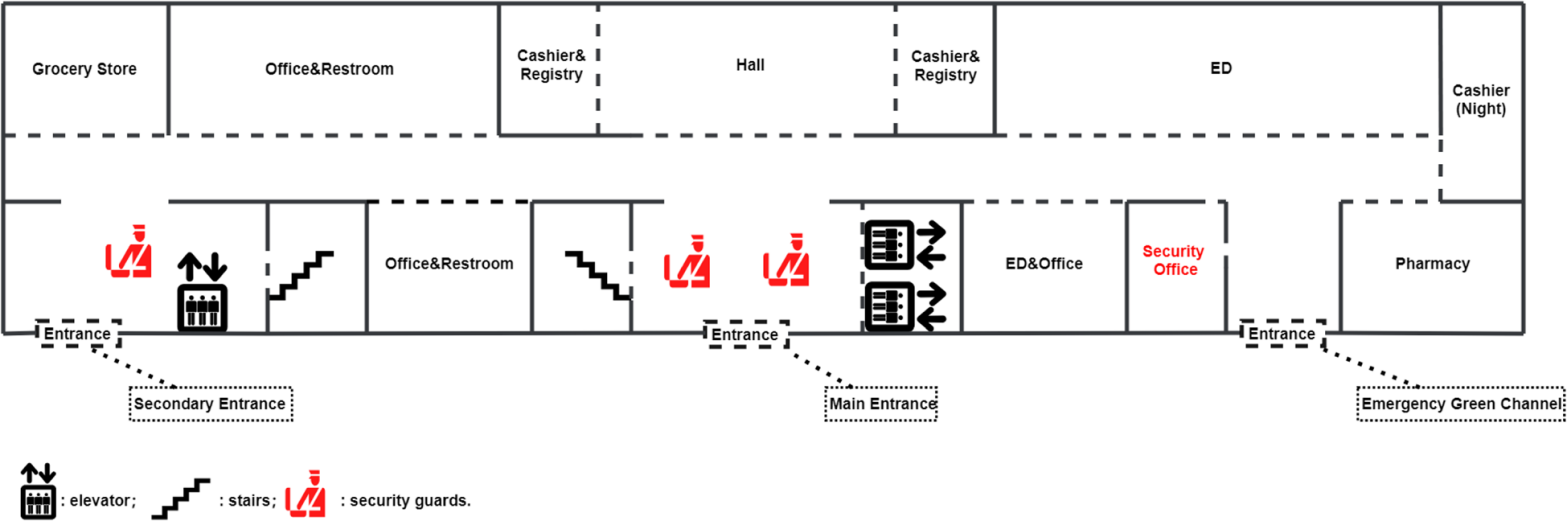
Layout of the Ground Floor

**Fig. 2:**
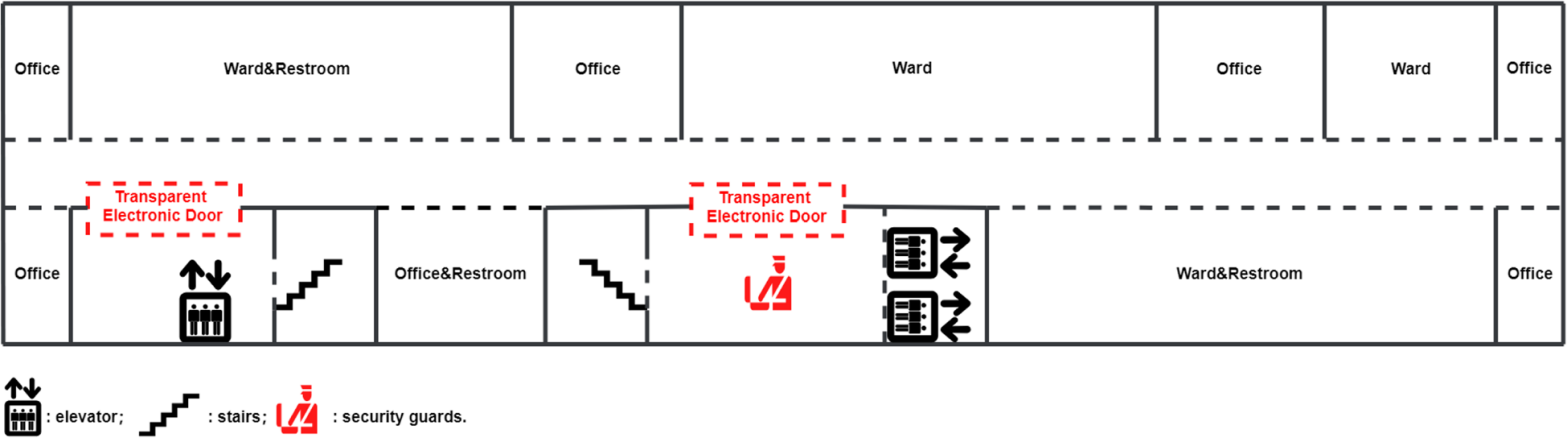
Layout above the Ground Floor

Generally speaking, the policy includes two parts: adding security guards and installing transparent electric doors. The emergency department (ED), cashier, registry, pharmacy, and a hallway are on the ground floor, and there are two female security guards at the main entrance and one at a secondary entrance. (Figure 1) There is no transparent electric door on the ground floor. On the other floors, the layout is almost the same: one transparent electric door at each entrance to ward, and one female security guard at the main entrance. (Figure 2) Visitors can ask healthcare workers in the ward to open the transparent electric door by using an electronic doorbell, which is connected to healthcare worker’s office on the particular floor. Staff use electronic identity cards to open the doors. The cost of each transparent electric door is about 2500$. The salary of a security guard is 400 ~ 500$ per month. The maintenance cost is about 500$ per year.

There are rules for visitors and family members:

1. Visiting hours:6:30 a.m.- 7:30 a.m., 11:30 a.m. - 12:30 p.m., and 4:30 p.m. - 7:00 p.m. from Monday through Friday. 8:00 a.m.- 7:00 p.m. on weekends and national holidays.
2. Visitors need identification to apply for visiting cards to get into wards. No more than 2 visitors per patient per visit.
3. Organizations should apply for permission from the administration office before visiting.
4. Doctors and nurses have the right to reject visit requests for medical reasons.
5. Healthcare workers have the right to decide on the number of caregivers. There is no more than 1 principal caregiver. The principal caregiver must get a permission card.
6. Visitors and caregivers must follow healthcare professionals’ instructions.

### Questionnaire

#### Questionnaire for healthcare professionals

The questionnaire developed jointly by the International Labour Office (ILO), International Council of Nurses (ICN), World Health Organization (WHO), and Public Services International (PSI) in 2003 [26] was used by this study, after obtaining permission, translating, and modifying, to measure hospital WPV. We revised language according to hospital administers’ opinions. Next, 32 healthcare professionals from the hospital were selected to complete a two-week test-retest reliability test (0.86).

In S1 (see Additional files 1), the questionnaire included three parts: (1) demographics (e.g. gender, age, occupation); (2) experience of physical violence in the past 12 months (physical force against another person or group, that results in physical, sexual or psychological harm. i.e. beating, kicking, slapping, stabbing, shooting, pushing, biting, and pinching); and, (3) experience of psychological violence in the past 12 months (intentional use of power, including threat of physical force, against another person or group, that can result in harm to physical, mental, spiritual, moral, or social development. i.e. verbal abuse, threatening events, and sexual harassment). In S2 (see Additional files 2), based on the questionnaire in S1, we added items regarding healthcare professionals’ feelings about changes resulting from restricting access. We also selected 36 employees to conduct a two-week test-retest reliability test (0.83) for S2.

#### Questionnaire for security guards

We used a self-design questionnaire to investigate WPV against security guards, which included simple questions covering these topics: experience of psychological/physical violence in the past 12 months, the frequency of psychological/physical violence, the cause of WPV, and support after experiencing psychological/physical violence. (see Additional files 3).

### Data collection

#### Healthcare professionals

We asked for the consent from administration department to conduct investigation. There were 723 healthcare professionals in total when S1 was conducted in April 2017, including doctors, nurses and medical technicians. We excluded 271 healthcare workers including: (1) who didn’t perform their daily work in inpatient ward building; (2) whose work experience was less than 12 months; (3) who were absent during the survey. Besides, 17 healthcare professionals refused to participate in our investigation. Finally, 435 participants filled in the questionnaire. A total of 345 valid questionnaires (no missing value and logic error) were collected, and the effective response rate was 79.31%.

**Fig. 3:**
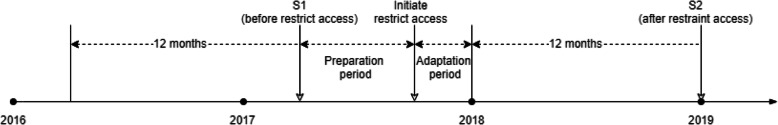
Timeline of Data Collection

There were 729 healthcare professionals (doctors, nurses and medical technicians) in total when S2 was conducted in January 2019. In S2, the exclusion criteria (1) and (3) was same with that of S1, except (2) was changed to: whose work experience was less than 24 months (these employees had not worked both before and after the policy was in place). According to the criteria, 312 healthcare professionals were excluded. Since 11 employees refused to participate in the survey, we investigated 406 healthcare professionals and collected 338 valid questionnaires (no missing value and logic error) in S2. The effective response rate was 83.25%. Figure 3 showed the timeline of data collection.

#### Security guards

The investigation towards security guards was conducted together with S2. We contacted with the manager of security guards and got the permission to investigate. At the beginning of the policy, there were 19 female security guards hired in total. 1 security guard left office for her own reasons in 2018. Finally, 18 female security guards were included and finished the questionnaire.

The data collection was anonymous and on the basis of voluntary. All the respondents, including healthcare professionals and security guards, were provided with informed consent, which described the purpose and method of data collection and kept the data confidential. The questionnaires were finished in a week when participants were free.

### Statistical analysis

Descriptive statistics analysis was used to summarize the demographic characteristics and prevalence of physical and psychological violence. Fisher’s exact test were used to compare prevalence of WPV and injury caused by WPV in 2 surveys. The data were entered by Epidata 3.1 and analysed by R version 3.6.0. The statistical significance level was set at 0.05.

## Results

Table 1 shows the demographic details of S1 and S2. Proportion of male and female in S1 was 28.70 and 71.30%, in S2 was 26.04 and 73.96%. There were 139 doctors (40.29%), 133 nurses (38.55%), 73 medical technicians (21.16%) in S1, 131 doctors (38.76%), 132 nurses (39.05%), 75 medical technicians (22.19%) in S2.

**Table 1:** Respondents’ Characteristic in S1 and S2

		S1 (*N* = 345)		S2 (*N* = 338)	
		*n*	%	*n*	%
Gender	Male	99	28.70	88	26.04
	Female	246	71.30	250	73.96
Age	≤30	130	37.68	130	38.46
	31–40	120	34.78	117	34.62
	41–50	78	22.61	68	20.12
	≥51	17	4.93	23	6.80
Profession	Doctor	139	40.29	131	38.76
	Nurse	133	38.55	132	39.05
	Medical technicians	73	21.16	75	22.19
Department	Internal medical	63	18.26	63	18.64
	Surgery department	51	14.77	57	16.86
	Obstetrics & Gynaecology	30	8.70	23	6.80
	Emergency department	28	8.11	25	7.40
	ICU	10	2.90	13	3.85
	Paediatrics	29	8.41	24	7.10
	Facial features	31	8.99	31	9.17
	Medical technology	73	21.16	75	22.19
	Others	30	8.70	27	7.99

The prevalence of psychological violence in S1 and S2 was 62.03 and 34.62%, that of physical violence was 3.77 and 4.73%, respectively. There was statistical significance in the prevalence of psychological violence(*P* = 0.000), but not of physical violence(*P* = 0.573) (Table 2). The rate of injury caused by physical violence was 76.92 and 31.25% in S1 and S2, respectively and Fisher’s exact test shows that the difference was statistically significant (*P* = 0.025). (Table 3).

**Table 2:** Fisher’s Exact Test of Prevalence of WPV in S1 and S2

	S1 (N^*^ = 345)		S2 (N^*^ = 338)		*P*
	*n*	%	*n*	%	
Psychological violence	214	62.03	117	34.62	0.000
Physical violence	13	3.77	16	4.73	0.573

*N: the number of respondents in S1 and S2 respectively

**Table 3:** Fisher’s Exact Test of Injury Caused by physical violence in S1 and S2

	S1 (n^*^ = 13)		S2 (n^*^ = 16)		*P*
	*n*	%	*n*	%	
Injured	10	76.92	5	31.25	0.025
Not Injured	3	23.08	11	68.75	

*n: the number of respondents who reported experience of physical violence in S1 and S2

Table 4 shows the prevalence of WPV in different professions and departments in S1 and S2.

**Table 4:** Fisher’s Exact Test of Prevalence of WPV of Different Professions and Departments in S1 and S2

	S1(n^*^ = 214)		S2(n^*^ = 117)		*P*
	*n*	%	*n*	%	
**Professions**					
Doctors	73	52.52	44	33.59	0.002
Nurses	104	78.20	54	40.91	0.000
Medical technicians	37	51.39	19	25.33	0.002
**Departments**					
Internal medical	46	73.02	25	39.69	0.000
Surgery department	45	88.24	25	49.12	0.000
Obstetrics & Gynaecology	10	33.33	4	17.39	0.225
Emergency department	19	67.86	15	60.00	0.580
ICU	4	40.00	0	0	0.024
Paediatrics	19	65.52	13	41.94	0.573
Facial features	18	58.06	8	33.33	0.020
Medical technology	37	51.39	19	25.33	0.002
Others	16	53.33	8	29.63	0.107

*n: the number of respondents who reported experience psychological violence in S1 and S2

When stratified by professions, doctors (*P* = 0.000), nurses(*P* = 0.000) and medical technicians (*P* = 0.002) suffered less psychological violence after the restricted access policy, which is statistically significant. As for the classification of clinical departments, the prevalence in internal medical (*P* = 0.000), surgery (*P* = 0.000), ICU (*P* = 0.025), facial features(*P* = 0.020) and medical technology (*P* = 0.002) show statistical significance.

Table 5 shows the respondents’ feelings about changes resulting from the restricted access policy. Only respondents of S2 answered these questions. Of those who responded, 84.32% felt that the medical order in wards was better, 82.54% thought that the restricted access policy helped enhance their sense of security, and 74.26% perceived that there was more attention from the organization with the policy. About 75% thought that it was in reducing their anxiety about WPV. However, less than 70% believed this policy strengthened their confidence in dealing with WPV, and, participants felt that it had a negative impact on WPV against security guards (20.12% for psychological violence, 13.31% for physical violence). Approximately 33% were worried about the restricted access policy causing visitor dissatisfaction.

**Table 5:** Respondents’ Feelings about Changes Resulting from the Restricted Access Policy

	Worse		No change		Better	
	*n*	%	*n*	%	*n*	%
Medical order in wards	7	2.07	46	13.61	285	84.32
Sense of security of healthcare workers	5	1.48	54	15.98	279	82.54
Attention from organization	13	3.85	74	21.89	251	74.26
Anxiety level on WPV	10	2.96	78	23.08	250	73.96
Confidence in dealing with WPV	4	1.18	102	30.18	232	68.64
Psychological violence towards security guards	68	20.12	69	20.41	201	59.47
Physical violence towards security guards	45	13.31	81	23.96	202	59.76
Satisfaction of visitors	113	33.43	52	15.38	173	51.18

All of the security guards in this hospital completed the questionnaire for security guards. The security guards were all female (*N* = 18). The prevalence of psychological violence and physical violence towards security guards was 88.89 and 33.33%, respectively. Half of the victims of psychological violence reported that the frequency was six times or more. ‘Visit request was rejected’ was the main reason for both psychological violence (62.25%) and physical violence (66.66%). Colleagues provided greatest support to victims. (Table 6).

**Table 6:** WPV towards Security Guards

		Psychological violence (n^*^ = 16,88.89%)		Physical violence (n^*^ = 6, 33.33%)	
		*n*	%	*n*	%
Frequency	1	3	18.75	5	83.33
	2–5	5	31.25	1	16.67
	≥6	8	50.00	0	0
Reason	Visit request was rejected	10	62.50	4	66.66
	Perpetrator was drunk	2	12.50	1	16.67
	Perpetrator was restless	4	25.00	1	16.67
Support after WPV	Comfort form leader	3	18.75	1	16.67
	Comfort from colleague	9	56.25	3	50.00
	Time for rest	0	0	1	16.67
	Comfort from family	1	6.25	0	0
	Comfort from friends	3	18.75	1	16.66

*n: security guards who reported experience of WPV

## Discussion

This paper studied WPV before and after implementation of a restricted access policy in a Chinese tertiary hospital. Compared to our previous study, the prevalence of physical violence was slightly lower both before and after the restricted access policy [27–31]. We speculated that may be due to the situation of investigation hospital. In China, sudden deterioration of patients’ condition [32], treatment outcome not meet the expectation [33, 34], waiting for a long time to receive healthcare services [35] were main reasons of WPV perpetration. In our study, the investigation hospital is not the most capable general hospitals in this province. Patients who were in severe conditions or wanted to achieve a better treatment outcome would be transferred to other hospitals with higher level medical service, which reduced the chance that healthcare professionals might be blamed for the patient’s deterioration. In addition, the number of patients who came to seek medical service was not too much to lead to long waiting time, which was also a reason that the prevalence of physical violence is lower than previous studies both in S1 and S2.

Our results indicate that the reported prevalence of psychological violence varied to a statistically significant degree from before to after the policy was implemented. Due to limited relative research, it is hard to compare the results with others. We speculate that there are several reasons for the difference: (1) the restricted access policy may have prevented inappropriate people from getting inside wards, which also has could have reduced potential perpetrators; (2) Transparent electric doors and security guards may deter the visitors or caregivers who may take a violent action; (3) In recent years, China has enacted laws and regulations as WPV intervention in social level, which may have contributed to a WPV decrease. As for physical violence, we did not see a reduction in prevalence, but the injury rate decreased from S1 to S2 at a rate which has statistical significance. This may mean the severity of the consequences of physical violence have reduced. Consistent with previous studies, environmental design would not necessarily reduce the prevalence of physical violence directly, but may be effective on reducing the severity of assaults [8, 36]. We assume the following circumstances could explain this finding in our study: (1) restricted access decreased the number of violent companions in the ward; (2) security may have prevented escalation to the point of injury when physical violence did occur. In the future, attention should be given not only to the prevalence of WPV, but also the extent to which control of WPV can reduce severe consequences after intervention measures have been implemented.

Regarding psychological violence, doctors, nurses and medical technicians all suffered less psychological violence after the implementation of restricted access policy, which means that the restricted policy may affect WPV occurrence with no difference in each profession. Previous study has shown that wards and offices are the high-risk place for WPV occurrence [16]. Since family accompany is Chinese tradition when someone was sick, restricted access policy has limited the number of companions when patients were receiving healthcare services in doctors’ office, wards or examination room, which may contribute to the WPV reduction in these three professions. Prevalence in different departments decreased from S1 to S2, but the differences are statistically significant only in the internal medicine, surgery department, ICU, facial features and medical technology departments. Meanwhile, ED, [37, 38] paediatrics, [39, 40], which are WPV high-risk departments, showed no statistically significant change in our study. Further research is needed to verify this finding and determine why this may be true.

Previous research suggested that female security guards are good at utilizing verbal skills, which may be instrumental in avoiding WPV [41]. Additionally, as the proverb goes ‘gentlemen don’t fight women’. These are the reasons why the hospital hired female security guards. However, the WPV prevalence towards security guards was high. The implementation of the restricted access policy may lead to more WPV against security guards. We suggest that when a restricted access policy is in place, security guards become scapegoats for the violence that would have been directed at healthcare professionals, thus resulting in guards facing more frequent and concentrated WPV. The experience of WPV was associated with a higher burnout score [42] among security guards, which may influence their work performance under the restricted access policy, thus leading to the rebound of WPV towards healthcare professionals. Accordingly, it is essential to improve this policy or to provide solid support for security guards, reducing WPV altogether rather than transferring it to different victim groups.

In the opinion of the healthcare professionals in our study, the restricted access policy is good for medical order in wards, which may be due to the decrease in outsiders and caregivers alleviating crowding and chaos in the wards. Research has suggested that security features which are easily noticeable could enhance the perception of safety [43]. In our study, healthcare professionals felt safer under the policy than before, which may due to the transparent electric doors and security guards being visible to them in their daily work. The activation of this policy also indicates more attention and support from organization. A 2004 study noted that having trust and a fair work environment positively influence the reduction of WPV [44]. Having a high level of organizational support is also effective in reducing tension and stress for those who have experienced WPV [45]. Previous studies also suggested that a higher anxiety level regarding WPV increases the odds of experiencing it [27, 28]. According to the cyclical model by Whittington and Wykes, the pressure caused by WPV can lead to adaptive behaviours that might create opportunities for violence to recur [46]. In our study, healthcare professionals thought that alleviating their anxiety about WPV with the restricted access policy may a positively affect the reduction of WPV. Moreover, this policy has helped healthcare professionals enhance their confidence in managing WPV. Although restricted access has a positive impact on WPV, it brings about some problems as well. Not including the WPV towards security guards as mentioned before, the dissatisfaction of visitors perceived by healthcare professionals is also a potential risk, which could be because this policy breaks the habit of families and friends accompanying or visiting patients. This may deteriorate the doctor-patient relationship thus leading to WPV towards both healthcare professionals and security guards in future.

### Limitations

This study has a few limitations. First, due to recall bias or reporting bias resulting from shame and stigma, WPV might not be accurately reported. Second, our study evaluated the impact of a restricted access policy on WPV in a tertiary general hospital, which might not adequately represent all general hospitals. Third, there may be some other factors could explain the changes of WPV, which is difficult to quantify and take into analysis. In addition, there may be honeymoon period after the introduction of the policy distorts the result, next we will follow the effectiveness of this policy on WPV. However limited, the study did provide some potentially important data and insights. Our study is innovative in focusing in on the impact of a restricted access policy on WPV, which may provide some direction and inspiration for future study or a point of reference for hospitals planning to carry out this policy.

## Conclusion

This research explored the restricted access impact on WPV in hospital. The results indicate that it may have positive impact on the prevalence and severity of WPV as well as how healthcare professionals perceive WPV. However, it aggravates WPV towards security guards and may decrease the satisfaction of visitors. As a rigid intervention to restrict people’s close interactions with healthcare professionals, the restricted access policy may not fundamentally improve doctor-patient relationships. Although the policy seems to have some positive effects so far, it may accumulate dissatisfaction and lead to future outbreaks of WPV. Review of our study may suggest that restricted access may not be a permanent solution or the most effective measure of intervention for WPV in hospitals. In addition to being able to examine the effectiveness of the policy as discussed in our study, other hospitals could use the data provided as a reference regarding the costs of implementing a restricted access policy.

## Supplementary information

**Additional file 1.** Investigation on hospital workplace violence for medical workers

**Additional file 2.** Investigation of Workplace violence for medical workers under restricted access policy

**Additional file 3.** Investigation on hospital workplace violence for security guards

## Data Availability

The data that support the findings of this study are available from Harbin Medical University and investigation hospital but restrictions apply to the availability of these data, which were used under license for the current study, and so are not publicly available. Data are however available from the authors upon reasonable request and with permission of Harbin Medical University and investigation hospital.

## Abbreviations

WPV: Workplace violence
OSHA: Occupational Safety and Health Administration
ED: Emergency department
ILO: International Labour Office
ICN: International Council of Nurses
WHO: World Health Organization
PSI: Public Services International
