# Supplementary material for: What is the impact of restricted access policy on workplace violence in general hospital? A before-after study in a CHINESE tertiary hospital
Source: BMC Health Serv Res. 2020 Oct 12;20:936. doi: 10.1186/s12913-020-05757-7 (PMC7549238; doi:10.1186/s12913-020-05757-7)
Supplement: Supplementary file 1 — Additional file 1. Investigation on hospital workplace violence for medical workers [file 12913_2020_5757_MOESM1_ESM.pdf]

Investigation on hospital workplace violence for medical workers

**Part 1: Demographic**	
**Gender:** ☐Male ☐Female	
**Age:**	
**Marriage status:** ☐Single ☐Married ☐Divorced ☐Widowed	
**Ethnicity:**	
**Education background:**<br>☐High school ☐Vocational school ☐Undergraduate ☐Master ☐PhD	
**Title:** ☐Senior title ☐Middle title ☐Junior title ☐No title	
**Profession**	☐Doctor ☐Nurse ☐Medical technician
**Work place in last 12 months**	☐Inpatient ward<br>☐Outpatient building<br>☐Other places
**Department**	☐Internal medical<br>☐Surgery department<br>☐Obstetrics & Gynaecology<br>☐Emergency department<br>☐ICU<br>☐Paediatrics<br>☐Facial features<br>☐Medical technology<br>☐Others
**You have worked in this hospital since__________ (month, year)**	
**Part 2: Physical violence**<br>*(physical force against another person or group, that results in physical, sexual or psychological harm. i.e. beating, kicking, slapping, stabbing, shooting, pushing, biting, and pinching)*	
**Have you ever experienced physical violence in last 12 months?**	
☐Yes ☐No (jump to Part 3)	
**Are you injured from of physical violence**	
☐Yes ☐No	
**Part 3: Psychological violence**<br>*(intentional use of power, including threat of physical force, against another person or group, that can result in harm to physical, mental, spiritual, moral, or social development. i.e. verbal abuse, threatening events, and sexual harassment)*	
**Have you ever experienced psychological violence in last 12 months?**	
☐Yes ☐No	
