# Supplementary material for: What is the impact of restricted access policy on workplace violence in general hospital? A before-after study in a CHINESE tertiary hospital
Source: BMC Health Serv Res. 2020 Oct 12;20:936. doi: 10.1186/s12913-020-05757-7 (PMC7549238; doi:10.1186/s12913-020-05757-7)
Supplement: Supplementary file 2 — Additional file 2. Investigation of Workplace violence for medical workers under restricted access policy [file 12913_2020_5757_MOESM2_ESM.pdf]

# Investigation of Workplace violence for medical workers under restricted access policy

| Part 1: Demographic | |
|---|---|
| **Gender:** □Male □Female | |
| **Age:** | |
| **Marriage status:** □Single □Married □Divorced □Widowed | |
| **Ethnicity:** | |
| **Education background:**<br>□High school □Vocational school □Undergraduate □Master □PhD | |
| **Title:** □Senior title □Middle title □Junior title □No title | |
| **Profession** | □Doctor □Nurse □Medical technician |
| **Work place in last 12 months** | □inpatient ward<br>□outpatient building<br>□other places |
| **Department** | □Internal medical<br>□Surgery department<br>□Obstetrics & Gynecology<br>□Emergency department<br>□ICU<br>□Pediatrics<br>□Facial features<br>□Medical technology<br>□Others |
| **You have worked in this hospital since______ (year)** | |
| **Part 2: Physical violence**<br>*(physical force against another person or group, that results in physical, sexual or psychological harm. i.e. beating, kicking, slapping, stabbing, shooting, pushing, biting, and pinching)* | |
| **Have you ever experienced physical violence in last 12 months?** | |
| □Yes □No (jump to Part 3) | |
| **Are you injured from of physical violence** | |
| □Yes □No | |
| **Part 3: Psychological violence**<br>*(intentional use of power, including threat of physical force, against another person or group, that can result in harm to physical, mental, spiritual, moral, or social development. i.e. verbal abuse, threatening events, and sexual harassment)* | |
| **Have you ever experienced psychological violence in last 12 months?** | |

| | | | |
|---|---|---|---|
| □Yes □No (jump to Part 4) | | | |
| **Part 4: Your Feelings about Changes Resulting from the Restricted Access Policy** | | | |
| | Worse | No change | Better |
| Medical order in wards | | | |
| Sense of security of healthcare workers | | | |
| Attention from organization | | | |
| Anxiety level on WPV | | | |
| Confidence in dealing with WPV | | | |
| Psychological violence towards security guards | | | |
| Physical violence towards security guards | | | |
| Satisfaction of visitors | | | |
