# Supplementary material for: What is the impact of restricted access policy on workplace violence in general hospital? A before-after study in a CHINESE tertiary hospital
Source: BMC Health Serv Res. 2020 Oct 12;20:936. doi: 10.1186/s12913-020-05757-7 (PMC7549238; doi:10.1186/s12913-020-05757-7)
Supplement: Supplementary file 3 — Additional file 3. Investigation on hospital workplace violence for security guards [file 12913_2020_5757_MOESM3_ESM.pdf]

Investigation on hospital workplace violence for security guards

Part 1: Demographic
**Gender:**
**Age:**
**Marriage status:** □Single □Married □Divorced □Widowed
**Ethnicity:**
**Education background:**<br>□High school □Vocational school □Undergraduate □Master □PhD
**Part 2: Physical violence**<br>*(physical force against another person or group, that results in physical, sexual or psychological harm. i.e. beating, kicking, slapping, stabbing, shooting, pushing, biting, and pinching)*
**Have you ever experienced physical violence in last 12 months?**
□Yes, frequency: _____ □No (jump to Part 3)
**Reason of physical violence occurrence: _______________**
**Support you had after physical violence occurrence:**
□Comfort form leader<br>□Comfort from colleague<br>□Time for rest<br>□Comfort from family<br>□Comfort from friends
**Part 3: Psychological violence**<br>*(intentional use of power, including threat of physical force, against another person or group, that can result in harm to physical, mental, spiritual, moral, or social development. i.e. verbal abuse, threatening events, and sexual harassment)*
**Have you ever experienced psychological violence in last 12 months?**
□Yes, frequency: _____ □No
**Reason of psychological violence occurrence: _______________**
**Support you had after psychological violence occurrence:**
□Comfort form leader<br>□Comfort from colleague<br>□Time for rest<br>□Comfort from family<br>□Comfort from friends
